# Microinjection Manipulation Resulted in the Increased Apoptosis of Spermatocytes in Testes from Intracytoplasmic Sperm Injection (ICSI) Derived Mice

**DOI:** 10.1371/journal.pone.0022172

**Published:** 2011-07-20

**Authors:** Yang Yu, Chun Zhao, Zhuo Lv, Wen Chen, Man Tong, Xuejiang Guo, Liu Wang, Jiayin Liu, Zuomin Zhou, Hui Zhu, Qi Zhou, Jiahao Sha

**Affiliations:** 1 State Key Laboratory of Reproductive Biology, Institute of Zoology, the Chinese Academy of Science, Beijing, China; 2 State Key Laboratory of Reproductive Medicine, Department of Histology and Embryology, Nanjing Medical University, Nanjing, China; 3 Graduate School of Chinese Academy of Sciences, Beijing, China; Institute of Zoology, Chinese Academy of Sciences, China

## Abstract

The invention of intracytoplasmic sperm injection (ICSI) has possibly been the most important development in reproductive medicine, one that has given hope to thousands of infertile couples worldwide. However, concerns remain regarding the safety of this method since it is a more invasive procedure than *in vitro* fertilization (IVF), since a spermatozoon is injected into the oocyte cytoplasm. Using mice derived from IVF technology as a control, we assessed the influence of invasive microinjection in the process of transferring sperm into oocyte cytoplasm in ICSI procedure on the development and physiologic function of resultant offspring. Our results demonstrated that mice produced from ICSI and IVF had no significant difference in phenotypic indices including body weight, forelimb physiology, and learning and memory ability. However, increased spermatocyte apoptosis was observed in the testis of adult ICSI mice, when compared with IVF mice. And, decreased testis weight and marked damage of spermatogenic epithelia were found in aged ICSI mice. Furthermore, proteomic analysis verified that most of the differentiated proteins in testes between adult ICSI and IVF mice were those involved in regulation of apoptosis pathways. Our results demonstrated that the microinjection manipulation used in the ICSI procedure might pose potential risks to the fertility of male offspring. The changed expression of a series of proteins relating to apoptosis or proliferation might contribute to it. Further studies are necessary to better understand all the risks of ICSI.

## Introduction

The treatment of human infertility by assisted reproductive technologies(ARTs)has gained widespread application, and the concerns on the risk of ARTs are also growing [1], [2]. Many studies have indicated there is an increased incidence of genetic, physical, or development abnormalities in ART offspring [3], [4]. However, most of these risk evaluations for ARTs were achieved by population-based cohort study [5]–[7], which could not distinguish the influence between the infertile factors of parents and ART technology. Romundstad et al reported that adverse perinatal outcomes of assisted fertilisation might be attributable to the factors leading to infertility, rather than to factors related to ART technology [8]. Therefore, appropriate animal models may be a better tool for studying potential effects of ART technology itself on its offspring, which can even up the influence of other factors. Recently, several studies using animal models have been reported which suggest that the embryo culture and transfer may produce specific abnormalities during fetal and postnatal development [9], [10]. The effects of other ART technology are still not systematic studied.

Intracytoplasmic sperm injection (ICSI) has been one of the main clinical components of ARTs since 1992 [11]. It is being used more frequently to treat couples with severe infertility disorders, especially those with male-factor induced infertility [12], [13]. ICSI is a more invasive procedure than IVF (*in vitro* fertilization), since a spermatozoon is injected into the oocyte cytoplasm through the zona pellucida and membrane, thereby ensuring fertilization. Even without considering that the sperm used for ICSI could potentially carry genetic abnormalities or structural defects, theoretically the injection procedure itself could result in physical damage or biochemical disturbances in the oocyte. These risks would include disruption of subcellular compartments, introduction of foreign material into the oocyte, changes in intracellular ion concentrations, and circumvention of the natural selection processes [14]. From the outset, the invasive nature of ICSI has led to some concerns on its safety and the potential risks to the offspring conceived by ICSI.

In recent years, some studies have been reported which investigate the influence of ICSI on resulting offspring. Most of these studies are clinical investigations and can not solely assess the effect of ICSI technology itself [6], [15]–[17]. Caperton *et al.* using a mouse model compared the frequency and spectrum of point mutations in midgestation fetuses from naturally conceived mice and fetuses produced by ICSI, which indicated ICSI method did not influence the frequency or spectrum of *de novo* point mutations in its offspring [18]. The influence of ICSI technology, especially the invasive microinjection manipulation specifically used in ICSI, on postnatal development and physiology of resulting offspring is still unknown. Long-term and systematic examinations are necessary to conclude whether ICSI related manipulations are safe for the offspring.

In the present study, we constructed a mouse model of ICSI and used IVF mouse model as control, by which the potential risk of microinjection could be solely evaluated. Various indices of behavior, morphology, and molecules were compared between the ICSI and IVF offspring from birth to old age. Our results indicated microinjection manipulation used in ICSI might pose potential risks to the fertility of male offspring.

## Materials and Methods

### Ethics Statement

All experiments requiring the use of animals were approved by the Committee on the Ethics of Animal and Medicine of the Institute of Zoology, Chinese Academy of Sciences (Permit Number: 20080915) and the Committee on the Ethics of Animal Experiments of Nanjng Medical University (Permit Number: 20080325).

### Animals

ICR mice were utilized throughout. ICSI offspring means that the mice conceived from the ICSI embryos which were transferred into the oviduct of pseudo-pregnant female mice, these mice were designed as ICSI group. And the in-vitro fertilization (IVF) offspring means that the mice conceived from the IVF embryos which were transferred into the oviduct of pseudo-pregnant female mice, these mice were designed as IVF group, namely control group in the present study. ICSI and IVF mice of 10-weeks old were regarded as adult, and those ranging in age from 48∼86 weeks were regarded as aged.

### Reagents

All chemicals used to produce ICSI and IVF embryos were purchased from Sigma-Aldrich Chemical Co (St. Louis, MO, USA) unless otherwise stated. All embryo manipulations, from oocyte collection to embryo transfer, were performed at 37°C. All culture media were pre-incubated to 37°C in a humidified atmosphere of 5% CO_2_ incubator.

### Oocyte collection

Oocytes were obtained from adult ICR mice that were superovulated by injection of 10 IU of pregnant mare serum gonadotropin (Hua Fu Biotechnology Company, Tianjin, China) and 10 IU of human chorionic gonadotropin (hCG; Hua Fu Biotech. Co.) given 48 h apart. After collecting the oocytes from the oviducts 16 h after administration of hCG, the cumulus-oocyte complexes were separated into two fractions. The first fraction was cultured in CZB-culture medium [19] at 37°C and 5% CO_2_ and prepared for ICSI manipulation. The cumulus cells were removed by brief incubation in 100 U/ml hyaluronidase dissolved in CZB-HEPES medium before ICSI manipulation. In the second fraction, cumulus-oocyte complexes were put directly into CZB-culture medium at 37°C and 5% CO_2_ without any treatment, and cultured for 1 h before IVF manipulation.

### ICSI procedure

Sperm which were collected from the cauda epididymis of adult ICR mice were washed two times with injection buffer(75 mmol/L KCl and 20 mmol/L HEPES, pH 7.0), then treated with buffer containing 12% polyvinylpyrrolidone. Active sperm were delivered into the oocyte cytoplasm using a Piezo micropipette-driving unit for cloning as described by Kimura and Yanagimachi [20]. A slight piezoelectrical pulse was applied to puncture the oocyte plasma membrane following penetration of the zona pellucida. Mouse sperm heads were separated from tails by applying a few piezo-pulses at the mid-piece of the sperm, immediately prior to injection into the oocyte. Activation was assessed by the number of zygotes with extrusion of the second polar body and two pronuclei at 6 h post-ICSI, and cleavage to the 2-cell stage after 24 h. In vitro development of embryos was assessed by monitoring progression to the blastocyst stage on day 4.

### IVF procedure

Conventional IVF was conducted using human tubal fluid (HTF) medium. The collected sperm from the cauda epididymis of adult male ICR mice were suspended in HTF medium at least 30 seconds, and then placed the dish in incubator for capacitation. Capacitation of spermatozoa was achieved by allowing them to stay at 37°C under 5% CO_2_ and 95% humidity for 1–2 h. The preincubated, capacitated sperm suspension was gently added to the freshly ovulated cumulus-oocyte complexes to give a final motile sperm concentrationof 1∼2×10^6^/ml that was determined by a hemacytometer. Sperm and oocytes were co-cultured for 4–5 h in insemination medium, and oocytes were then cultured in CZB-culture medium and assessed for their development efficiency in vitro.

### Housing and Behavior

After ICSI or IVF manipulations, the embryos (2-cell stage) were transferred into the oviduct of pseudopregnant CD-1 females and delivered naturally by the pregnant mice on day 19.5. ICSI and IVF offspring were maintained under controlled temperature and lighting conditions, and given food and water adlibitum. Postnatal development of mice was evaluated according to a panel of physiological indices, including body weight, reproductive ability, forelimb force, and learning and memory ability. Mice in both groups were weighed weekly up to 10-weeks old, and once every two weeks thereafter until 80-weeks old. At 8∼12 weeks old, ten mice/sex/group were cross-mated between the two groups to examine their reproductive ability, and the next generation was delivered naturally by the pregnant mice on day 19.5.

To test forelimb physiology, a puller designed by our “Pin” method was used to test the muscle strength (force index) when the mice were 6 weeks old (six mice/sex/group). We connected the pins to each other as a chain, which the mice could clutch at. The mice were suspended inversely, and caught hold of the chain. Pins were added onto the chain until the mice discarded them by themselves. The weight of each chain was regarded as the forelimb strength of the mouse.

The learning and memory ability was assayed by using the Morris water maze test as described [21], [22]. In brief, a total of 36 trials were performed over 6 consecutive days. During acquisition trials performed over the first 3 days, the mice were expected to find the hidden platform located in the fourth quadrant as soon as possible within 60 s. The probe tests were then completed on the 4^th^ day, with the platform removed. To determine if the mice were selectively swimming in the quadrant in which the platform had previously been located (fourth quadrant), the times that the mouse spent in each of the 4 quadrants was measured. During the last 2 days, 12 reversal trials were administered, in which the platform was placed in the opposite quadrant at the same distance from the pool wall as in the first 3 days, and the times that the mouse found the platform were measured. The data were analyzed by tracking system software to evaluate learning and memory ability.

### Mouse tissue collection and weight

Tissue was collected at 10 weeks and 86 weeks from both ICSI and IVF mice. Mice were sacrificed and the viscera, as well as the thoracic and abdominal cavities were observed carefully. The key organs, including the heart, lung, liver, spleen, kidney, brain, testis and epididymis were then removed. Each organ was divided into two parts, one half was snap-frozen in liquid nitrogen for protein isolation and the other was fixed in 4% formaldehyde or bouin solution for histological examination. At 86-weeks old, each organ was weighed before being cut apart.

### Histology and histomorphometry analyses of the testis tissues

For these experiments, testes from adult and aged ART mice were fixed in bouin solution and embedded in paraffin. The blocks were then deparaffinized, sectioned (5 µm) and stained with hematoxylin and eosin for histological examination. The morphology of seminiferous tubules and germ cells were observed. 20 randomly selected visual fields and about 200 seminiferous tubules of each mouse were observed and counted, for determining the percentage of abnormal seminiferous tubules [23], [24]. The cellular morphology was carefully observed in approximately 250 seminiferous tubules for each animal.

The TUNEL assay for apoptotic cell detection was performed using the In Situ Cell Death Detection Kit (Boehringer Mannheim GmbH, Mannheim, Germany) and was performed according to the manufacturer's instructions. Apoptosis was visualized using anti-fluorescein antibody Fab fragments, conjugated with alkaline phosphatase (AP) and converter-AP. The numbers of TUNEL-positive cells in approximately 250 seminiferous tubules of each mouse were counted, and the apoptotic indices were then determined by calculating the ratio of total numbers of TUNEL-positive cells/numbers of counted seminiferous tubules.

### 2-DE, gel image analysis and protein identification

Proteins from the testes of three adult mice in each group were extracted and separated by 2-DE as previously reported [22]. Gels were silver stained, scanned and analyzed using ImageMasterTM 2D platinum software (Version 5.0, GE Healthcare, San Francisco, CA, USA). The expression level was determined by the relative volume of each spot in the gel and expressed as %Vol (%Vol = [spot volume/Σvolumes of all spots resolved in the gel]). We averaged the values from the three independent experiments of ICSI and IVF groups respectively, calculated the means and standard deviations and assessed the statistical significance with Student's t-tests using ImageMasterTM 2D platinum software. A spot was regarded significantly differentially expressed between groups if the average spot intensity was greater than 1.5-fold and P value by Student's t-test was also less than 0.05.

Protein spots with significant differences between the ICSI and IVF groups were excised. Gel pieces were denatured, alkylated, trypsin digested and analyzed by an Ultraflex II MALDI-TOF-TOF mass spectrometer (Bruker Daltonics GmbH, Bremen, Germany) under the control of FlexControlTM 2.4 software (Bruker Daltonics GmbH). The analysis and search conditions used for protein identification were the same as previously described [22].

### Pathway Analysis by PathwayStudio™

An analysis of cellular processes influenced by differentiated proteins in the ICSI testis compared with the IVF testis was performed using PathwayStudio™ (v5.0) software (Ariadne Genomics, Inc. Rockville, MA, USA). The text-mining software uses a database of molecular networks that are assembled from scientific abstracts and a manually created dictionary of synonyms to recognize biological terms [25]. The cellular processes influenced by the various treatments were determined by searching the database for the imported gene/protein and for the cellular processes in which the imported genes/proteins are involved. In our analysis, each identified cellular process was confirmed manually using the relevant PubMed/Medline hyperlinked abstracts.

### Western blotting

Proteins from 3 pairs of adult mouse testes per group (ICSI and IVF) were separated on 12% polyacrylamide gels and transferred to PVDF membranes (GE Healthcare, San Francisco, CA, USA). These blots were incubated for 1.5 h at room temperature in Tris-buffered saline (TBS) containing 5% nonfat milk powder. Primary antibodies used were anti-endoplasmic reticulum ERp57 polyclonal antibody (diluted 1∶1000, Abcam, USA), anti-β-tubulin polyclonal antibody (diluted 1∶2000, Abcam, USA), anti-GRP78 polyclonal antibody (diluted 1∶100, Santa Cruz, USA) and anti-heterogeneous nuclear ribonucleoprotein K (hnRNPK) polyclonal antibody (diluted 1∶500, Abcam, USA), anti-Omi monoclonal antibody (diluted 1∶500, Abcam, USA). Blots were incubated with primary antibodies overnight at 4°C. After washing 3 times in TBS, blots were incubated with horseradish peroxidase (HRP)-conjugated secondary antibody (1∶1000; Beijing ZhongShan Biotechnology CO., China) for 1 h. Specific proteins were detected using ECL reagents (GE Healthcare, San Francisco, CA, USA) and AlphaImager (FluorChem5500, Alpha Innotech, San Leandro, CA, USA). The protein expression level was analyzed by AlphaEaseFC software (Alpha Innotech, San Leandro, CA, USA).

### Immunohistochemistry staining

Bouin's solution-fixed paraffin-embedded sections from mouse adult testes were immunostained. After quenching the endogenous peroxidase activity, the sections were subjected to microwave antigen retrieval in 0.02 M EDTA. Thereafter, they were washed in PBS and blocked with goat or rabbit serum (Beijing ZhongShan Biotechnology Co., China) for 2 h. They were subsequently incubated overnight at 4°C with polyclonal ERp57 antibody (diluted 1∶1000, Abcam, USA), polyclonal GRP78 antibody (diluted 1∶50, Santa Cruz, USA), polyclonal hnRNPK antibody (diluted 1∶50, Abcam, USA), monoclonal Omi antibody (diluted 1∶50, Abcam, USA). After 3 washes in PBS, the sections were incubated with HRP-conjugated secondary antibody (1∶1000; Beijing ZhongShan Biotechnology CO., China) for 1 h at room temperature. Immunoreactivity was revealed with di-aminobenzadine (DAB; Sigma, Germany) for increased sensitivity, to produce a brown insoluble precipitate at immunopositive sites. Sections were counterstained with hematoxylin and mounted onto a cover glass. The negative controls were incubated with a solution devoid of any primary antibody.

### Statistics analysis

Data were analyzed by t-test for significant differences between the two groups. All ratios were arcsine square root transformed before t-test analysis and the least significant difference (LSD) post hoc test was used to examine any significant difference between groups. The results were considered statistically significant when p<0.05.

## Results

### Effects of microinjection manipulation on mouse embryo development

To examine the effects of microinjection manipulation used in ICSI on embryo development, we constructed the mouse model simulating the ICSI procedure used in human infertility treatment and used the IVF mice as a control. The development efficiency of derived embryos was compared between ICSI and IVF groups. As a result, we indicated that the development efficiency at the 2-cell stage, which is important in zygotic genome activation, did not differ between these two groups. However, there was a significant difference between the ICSI and IVF groups in later development of embryos. The development efficiency at the morula and blastocyst stages was significantly decreased in the ICSI group as compared with IVF group (P<0.05; Table 1). When 2-cell embryos were transferred into recipient females, nearly 64% of the embryos produced viable pups in the ICSI group and this result was comparable to the IVF group (P>0.05; Table 2).

**Table 1:** Development of embryos produced by the ICSI and IVF procedures *in vitro*.

Group	No. of embryos	No. of activated embryos (%)	No. of embryos develop to different stages (%)		
			2-cell	Morula	Blastocyst
ICSI	65	63 (96.9)^a^	63 (96.9)^a^	50 (76.9)^a^	47 (72.3)^a^
IVF	78	76 (97.4)^a^	76 (97.4)^a^	72 (92.3)^b^	69 (88.5)^b^

a–b Values with different superscripts in the same column are significantly different (P<0.05).

**Table 2:** Development of embryos produced by the ICSI and IVF procedures *in vivo*.

Group	No. of transferred embryos (2-cell)	No. of recipient females	No. of pregnant recipient females	No. of live pups (%)
ICSI	112	8	7	72 (64.2)^a^
IVF	86	7	7	61 (70.9)^a^

a Values with same superscripts in the same column are comparable (P>0.05).

### Phenotypes observed

Qualitative forelimb force was tested when the mice were six weeks of age. The results showed no differences between the ICSI and IVF groups within the same gender (Data not shown). Ten 10-week old mice of each sex were selected randomly from each group and tested for reproductive capacity. The results showed that all animals were fertile. The average number of pups generated from ICSI male mice (10.3±1.1) was lower than that generated from IVF male mice (13.7±2.4), but there were no significant differences between the two groups (P>0.05, Table 3).

**Table 3:** Reproductive capacity of mice produced from ICSI and IVF embryos.

Group	Gender	Reproductive ability	
		Mice examined (week old)	Average number of pups generated from male mice
ICSI	Male	10 (8∼12)	10.3±1.1^a^
	Female	10 (8∼12)	
IVF	Male	10 (8∼12)	13.7±2.4^a^
	Female	10 (8∼12)	

a Values with same superscripts in the same column are comparable (P>0.05).

**Figure 1:**
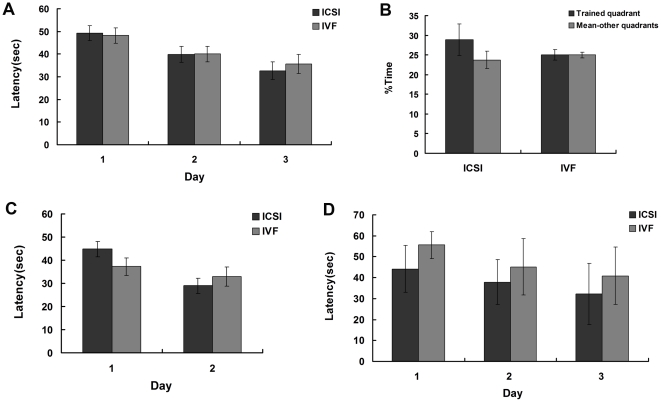
Results from the Morris water task showed there was no significant difference in acquisition trials, probe tests and reversal trials between ICSI and IVF groups (P>0.05). (A) Average latency to find hidden platform - acquisition trials (Adult mice). (B) Average time spent searching quadrant in which animal trained vs. other quadrants - probe tests (Adult mice). (C) Average latency to find hidden platform - reversal trials (Adult mice). (D) Average latency to find hidden platform - acquisition trials (Aged mice).

To study the spatial learning and memory ability of adult mice conceived by ICSI and IVF, a total of 36 trials were performed during 6 consecutive days by using a Morris water maze apparatus, on 10 mice (5/group). In acquisition trials, the results showed that both ICSI and IVF mice displayed evident improvement over the 3 days of testing, and the mean acquisition times in the two groups did not differ significantly from each other (P>0.05, Fig. 1A). In the probe tests, we found that the time that mice wandered in the fourth quadrant was longer than that in the other three quadrants, but there was no difference between the ICSI and IVF groups (P>0.05, Fig. 1B). In the reverse trials, the hidden platform was relocated into the opposite second quadrant, and the mice were retested. Both groups improved their performance over the 2 days of trials, with no significant difference observed between the two groups (P>0.05, Fig. 1C). Furthermore, to test whether the nervous function was influenced by ICSI manipulation with age, the Morris water maze was also performed in 24 (12/group)aged mice. In acquisition trials, the mean acquisition times also had no significant difference between the two groups (P>0.05, Fig. 1D). However, the aged mice failed to complete the probe trials and the reversal trials due to their poor health. Thus, the memory function could not be evaluated in the aged offspring.

### Body and organ weight

**Figure 2:**
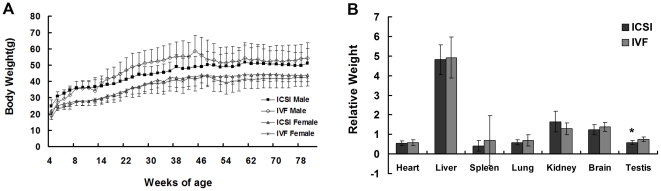
Body weight and organ weight. (A) Weight monitoring of mice (4–80 weeks old) showed no significant differences between the ICSI and control mice (p>0.05). (B) Examination of organ weight showed no significant difference in the weight of heart, liver, spleen, lung, kidney and brain between the ICSI and IVF groups, but the testis relative weight of ICSI mice was lower than that of IVF mice (* P<0.05).

The body weight of ISCI (female n = 7, male n = 6) and IVF (female n = 16, male n = 4) mice at 4∼80 weeks old was examined, and no remarkable differences were detected between these two groups (P>0.05, Fig. 2A). When mice were sacrificed at 86-weeks old, the important organs including heart, liver, spleen, lung, kidney, brain and testis were collected and their relative weight (percentage of organ weight relative to body weight) were analyzed. We found that there were no significant differences in organ weight between the ICSI (n = 11) and IVF (n = 18) groups, except that the testis relative weight of ICSI mice was lower than that of IVF mice (P<0.05; Fig. 2B).

### Histology analyses and apoptosis detection in testes

**Figure 3:**
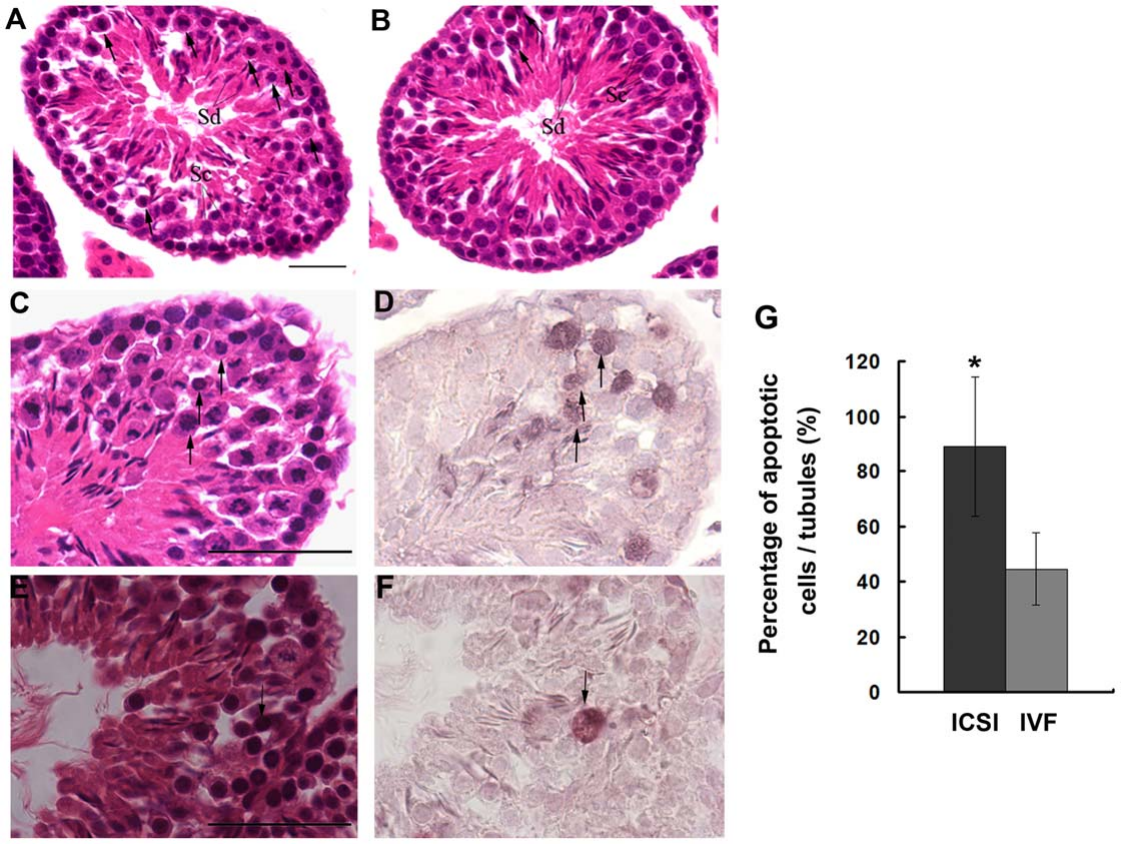
Testicular morphology of adult offspring derived from ICSI and IVF. The morphology of ICSI (A) and IVF testes (B) was examined by hematoxylin and eosin (HE) staining. Tubules in the ICSI testis contained more abnormal metaphase I and anaphase I spermatocytes in Stage XII (black arrows) compared to the IVF testis. Sc, spermatocyte; Sd, spermatid. HE staining showing many abnormal spermatocytes (black arrows) in the ICSI testis (C) and few abnormal spermatocytes (black arrows) in the IVF testis (E). (D, F) TUNEL staining demonstrating that these abnormal spermatocytes are apoptotic (black arrows). (G) The percentage of apoptotic spermatogenic cells in ICSI testes were much higher than that in IVF testes (*P<0.01). Bar: 20 µm.

Histological analysis was performed to determine why the relative weight of testes was decreased in the aged ICSI mice. The testis histology of adult mice (n = 6) was examined first. The results showed that although the morphology of seminiferous tubules in the ICSI mice appeared normal and similar to that in the IVF mice, many abnormal cells were observed in the seminiferous tubules of ICSI mice. In Stages X–XII of the adult ICSI testes, a large number of clustering metaphase I and anaphase I spermatocytes showing eosinophilic cytoplasm were frequently observed; and when these abnormal cells appeared, the above spermatids were rarely observed or decreased (Fig. 3A). This phenotype was less frequently occurring in the IVF offspring testes (n = 6, Fig. 3B). In addition, these cells were verified to be undergoing apoptosis, as assessed with the TUNEL assay (Fig. 3C, D, E, F). By counting the number of TUNEL-positive cells, we discovered that the percentage of apoptotic spermatogenic cells in ICSI testes was much higher than that in IVF testes (P<0.01, Fig. 3G).

**Figure 4:**
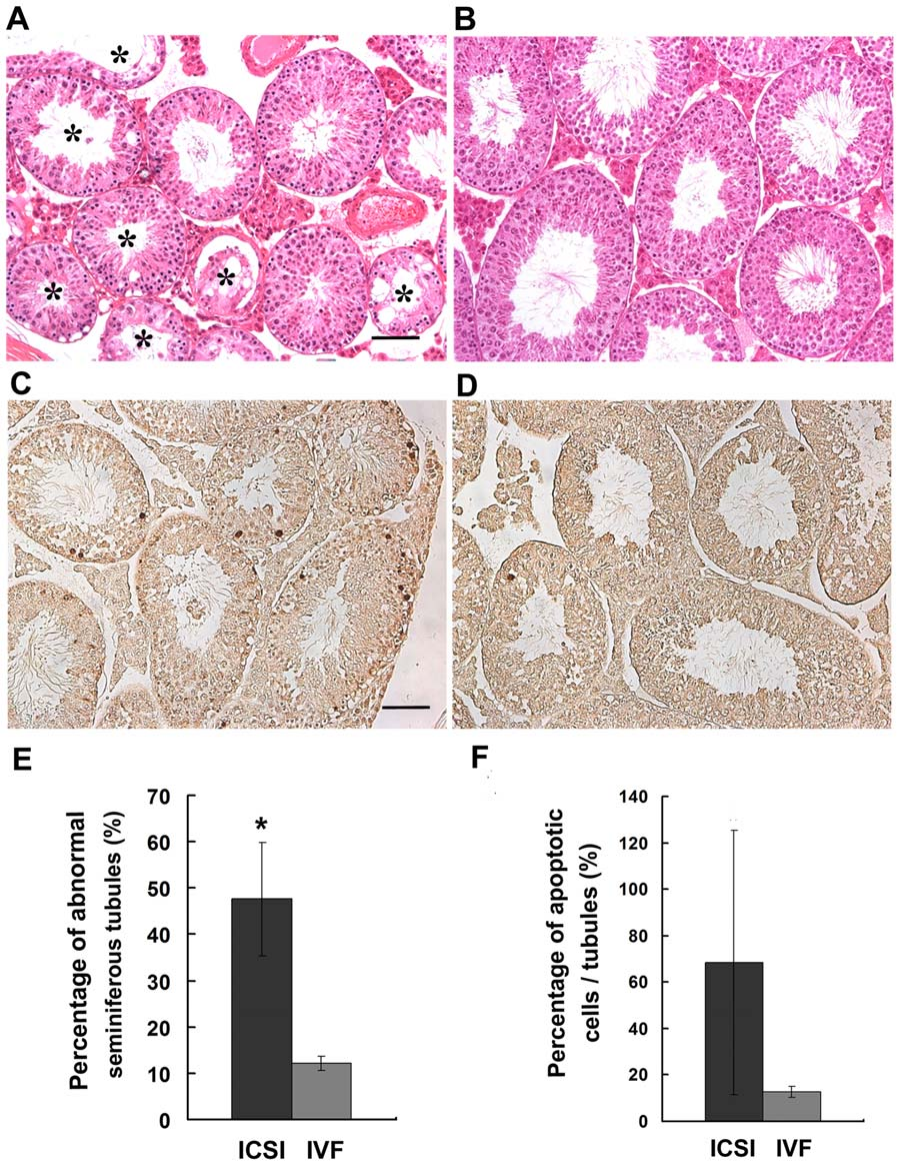
Testicular morphology of aged offspring derived from ICSI and IVF. The morphology of ICSI (A) and IVF testes (B) was examined by hematoxylin and eosin (HE) staining. The ICSI testis contained much more abnormal seminiferous tubule (*) than the IVF group. The TUNEL assay showed more spermatogenic cells undergoing apoptosis in aged ICSI testes (C) when compared to the IVF mice (D). (E) The percentage of seminiferous tubules with abnormal morphololgy in ICSI testes was significantly higher than that in IVF testes (* P<0.01). (F) The percentage of apoptotic spermatogenic cells in ICSI testes was comparable to the IVF group (P>0.05). Bar: 50 µm.

Furthermore, histological examination of testes from aged mice was performed. We found that the morphology of seminiferous tubules from IVF mice (n = 3) appeared normal (Fig. 4B). However, a large number of abnormal seminiferous tubules, including apparent premature release of germ cells into the seminiferous tubule lumen, vacuolization in the seminiferous tubules, and germ cell-deficient (e.g., Sertoli cell only) seminiferous tubules, etc. were observed in aged ICSI testes (n = 6, Fig. 4A). And, the percentage of seminiferous tubules with abnormal morphololgy in ICSI testes was significantly higher than that in IVF testes (P<0.01,Fig. 4E). The TUNEL assay showed more spermatogenic cells undergoing apoptosis in aged ICSI testes when compared to the IVF mice (Fig. 4C and D). However, statistical analysis showed the difference was not significant (Fig. 4F, P>0.05), probably because numerous spermatogenic cells had died in the aged ICSI mice.

### Analysis, identification and functional annotation of differentially expressed proteins in testes

**Figure 5:**
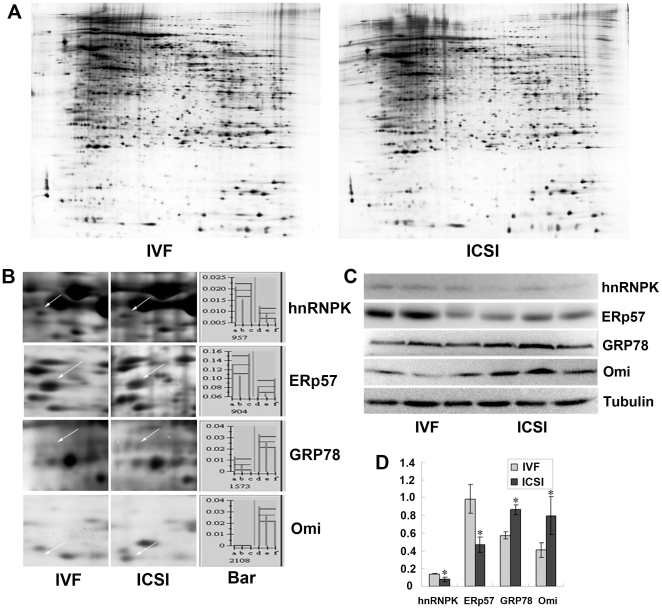
Results of Two-dimensional electrophoresis (2-DE) and validation wuth Western blotting. (A) 2-DE maps of proteins from IVF and ICSI testis. (B) Magnified comparison maps of spots for hnRNPK, ERp57, GRP78, and Omi in the 2-DE patterns of IVF and ICSI testis. The differentiated spots were indicated by arrows. Bar represents the relative volume (%vol) of each of the spots on the gels. (C) Western blot analysis with anti-hnRNPK, anti-ERp57, anti-GRP78, anti-Omi and anti-Tubulin antibodies were performed using 50–100 µg of total protein extracts prepared from three IVF testes and three ICSI testes. (D) Bars represent the density of gel bands determined from each of the three samples. Results are consistent with the results of the 2-DE.

The discernible damage in testicular histology had been noted in aged ICSI mice (Fig. 4A), which made the proteomic analyses of these testes unfeasible. Thus, only the testes from the adult groups were selected for proteomic analyses and used to elucidate the potential molecule mechanism behind the increased apoptosis of the spermatocytes. After 2-dimensional electrophoresis (2-DE), the gels from adult IVF and ICSI testes (Fig. 5A) were individually imaged and analyzed using ImageMaster™ 2D platinum software. 38 differentially expressed spots were found, and among these, 20 spots were significantly up-regulated in the ICSI group, while 18 spots were down-regulated. 25 spots were identified and further detailed information was listed in the Table S1.

To verify the 2-DE results, we selected 4 differentially expressed proteins, hnRNPK, ERp57, GRP78, and Omi for Western blotting analysis. As shown in Fig. 5C and D, levels of hnRNPK and ERp57 decreased and that of GRP78 and Omi increased in ICSI testes when compared to the control group. These results are consistent with those determined using the ImageMaster™ 2D Platinum Software (Fig. 5B).

**Figure 6:**
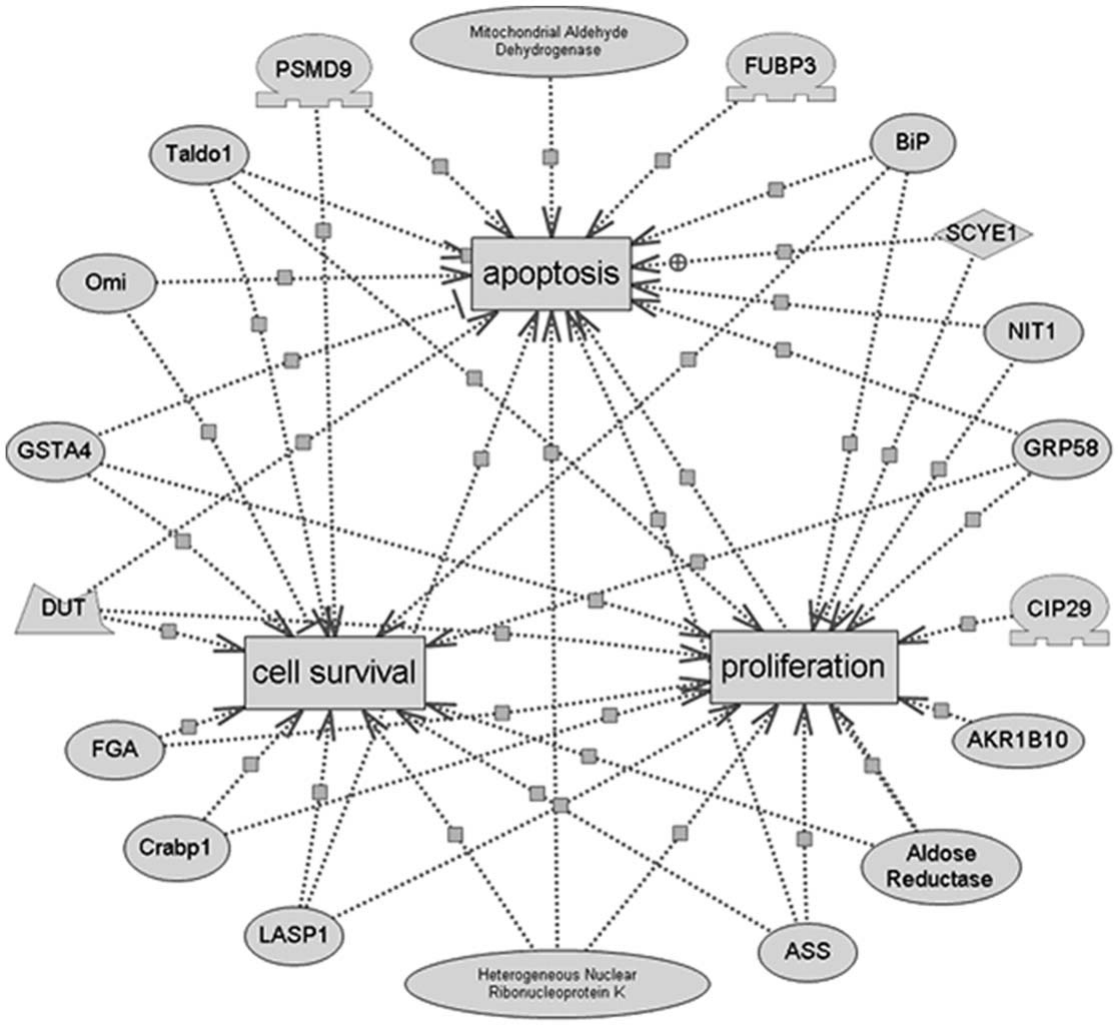
Regulated pathways involving the differentiated proteins in the ICSI testes compared with the IVF testes as predicted by PathwayStudio™ software. Oval-shaped signs represent proteins and cube-shaped signs represent cellular processes. Regulatory events are displayed with arrows and documented by literature citations.

To gain a better understanding of the 25 proteins identified in this study, a detailed analysis of the cellular processes influenced by these proteins was performed using PathwayStudio™ software. We found that most of the differentially-expressed proteins were involved in apoptosis (15 proteins), proliferation (15 proteins) or cell survival (13 proteins). The results are shown in more detailed graphs in Fig. 6.

### Localization of proteins in normal testis using immunohistochemical staining

**Figure 7:**
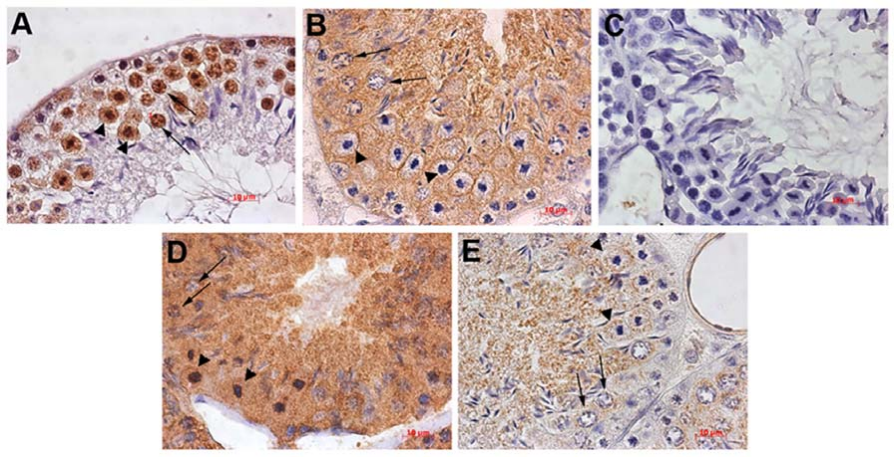
Immunolocalization of hnRNPK (A), ERp57 (B), GRP78 (D), Omi (E) and control antibody (C) in the normal adult mouse testis. Normal spermatocytes are indicated with arrows and the abnormal spermatocytes are indicated with arrow heads. Bar: 10 µm.

Immunohistochemical staining for hnRNPK, GRP78, ERp57 and Omi was performed on paraffin sections. The hnRNPK protein was expressed in the cytoplasm of spermatogonia, as well as in the nuclei of spermatocytes and round spermatid. It was also expressed in the cytoplasm and nuclei of the abnormal spermatocytes. Immunoreactivity for this protein in spermatogenic cells at later stages was almost completely absent (Fig. 7A). The ERp57 protein was expressed in the cytoplasm of all spermatogenic cells (Fig. 7B). Similarly, GRP78 was expressed in the cytoplasm of all spermatogenic cells, and was more abundant in the highly condensed nuclei of abnormal spermatocytes (Fig. 7D). Omi was diffusely expressed in the cytoplasm of normal and abnormal spermatocytes (Fig. 7E).

## Discussion

One of the key concerns on the use of ART is the safety of the procedures and consequences for long-term health. Since the invasive ICSI procedure is commonly used in ART clinics, it is necessary to investigate its safety and determine if it poses a risk to the resulting offspring, in terms of health outcomes and fertility [26].

Using the mouse model, a long-term evaluation on the potential risk of microinjection used in the ICSI procedure on resulting offspring was carried out in the present study. Our results demonstrated that the microinjection manipulation had no influence on the embryonic development and postnatal phenotypes of the ICSI offspring, including birth rate, body weight, forelimb physiology and learning and memory ability. However, we found that microinjection used in ICSI might pose potential risks to the reproductive physiology of male offspring. Histological analyses revealed that the testes derived from adult ICSI mice contained more abnormal metaphase I and anaphase I spermatocytes in Stage XII compared to the testes of IVF mice, and these abnormal spermatocytes were proven to be apoptotic cells as detected by TUNEL staining (Fig. 3). When ICSI mice were aged, it was found that their testes weight decreased and the structures of a large number of seminiferous tubules in the testes were markedly damaged (Fig. 4).

It is well known that apoptosis occurs spontaneously or inductively throughout mammalian spermatogenesis for the development of normal mature spermatozoa and for the elimination of excess or abnormal germ cells [27]. Apoptosis is commonly observed during spermatogenesis, especially at the spermatocyte stage [28], which is particularly sensitive to this process. It has been demonstrated that deregulation of apoptosis can lead to defective sperm formation and result in impaired spermatogenesis [27], [29]. In the present study, we found that the apoptosis of spermatogenic cells increased markedly in the ICSI adult testes as compared to the control mice, and the increased apoptotic cells finally caused the damage of spermatogenic epithelia when ICSI mice were old. This result was in accord with previous reports. Although a significant difference in reproductive ability between ICSI and IVF male adult mice was not observed, we have shown the average number of pups generated from ICSI male mice was lower than that generated from IVF male mice, in the present study. Furthermore, the more serious damage of testis structure observed in the aged mice implied that the reproductive ability of male ICSI mice might be destroyed gradually with age, and they might lose their reproductive ability earlier.

In order to clarify the potential mechanism behind the effect of microinjection on the germ cell apoptosis, we compared the protein expression profiles in adult testes of ICSI and IVF mice. The differentially expressed proteins resulting from the microinjection manipulation could help us understand the proteins influencing testicular development of ICSI offspring and provide further insight into their functions. A total of 38 protein spots exhibited significantly different expression patterns between the two groups and 25 proteins were successfully identified. We determined that 15 of these proteins were involved in regulation of the apoptosis pathway by bioinformatics analysis and these results were consistent with our histological findings (Fig. 3). More importantly, 13–15 of these proteins which were suggested to participate in the pathway of proliferation or cell survival, negatively regulate proliferation and cell survival in the ICSI offspring's testis, based on their changing expression patterns in the 2D gels of ICSI offspring testis compared to IVF offspring testis. The changed expression of the above proteins might contribute to the germ cell apoptosis and spermatogenic damage in the testis of ICSI offspring.

For example, hnRNPK is an evolutionarily conserved factor found in the nucleus and cytoplasm that has been implicated in cell cycle processing, chromatin remodeling and transcription, as well as mRNA splicing, export, and translation [30]. Previous studies have shown that down-regulation of hnRNPK in the somatic cells cause cell cycle arrest in response to DNA damage [31]. In contrast, overexpression of hnRNPK enhances breast cancer cell proliferation [32]. Here, we have shown that normal mouse testis tissue contains hnRNPK which is localized to the nuclei of primary spermatocytes and is diffusely expressed in the abnormal spermatocytes. Decreased expression of hnRNPK in the ICSI testis might inhibit the proliferation of spermatocytes and cause the cell to be apoptotic. ERp57, one of the best characterized protein disulfide isomerases, can regulate ER stress signaling [33]. In EZH2-knockdown cells, cell proliferation is inhibited and the expression of ERp57 is down-regulated [34]. Further studies have shown that knockdown of ERp57 down-regulates ER stress and enhances oxidative-stress induced apoptosis [35]. GRP78, also referred to as the immunoglobulin binding protein BiP, is another stress-inducible ER chaperone [36]. Some DNA damage activators will induce ER stress with increased expression of GRP78 [37]. In our study, both ERp57 and GRP78 were expressed in the spermatocytes with the decreased expression of ERp57 and increased expression of GRP78 in the ICSI testis. The changed expression pattern of these proteins in the spermatocytes can both induce the apoptosis of spermatocytes. Omi is a differentiated protein that is up-regulated in the ICSI testis. It is a nuclear-encoded protein that can be released from the mitochondria into the cytosol after an apoptotic stimulus to induce caspase-dependent and independent apoptosis [38]. Previous studies have also shown that overexpression of Omi causes apoptotic cell death [39], [40], which supports our current findings.

In conclusion, our results indicated that microinjection manipulation used in the ICSI procedure might pose potential risks to the fertility of male offspring. Although the direct mechanism by which microinjection acts on the testis is still unknown, the present results suggest that the changed expression of a series of proteins relating to apoptosis or proliferation might contribute to it. Disruptions of subcellular compartments, introducing foreign material into the oocyte, changes in intracellular ion concentrations brought by the microinjection manipulation might affect the expression of these proteins. Thus, it is necessary that an invasive technology, such as ICSI, must undergo further and full evaluation.

## Supporting Information

Table S1 Full peptide data sets.
(XLS)
